# Alterations in brain white matter microstructural properties in patients with Crohn’s disease in remission

**DOI:** 10.1038/s41598-020-59098-w

**Published:** 2020-02-07

**Authors:** Jiancheng Hou, Keith Dodd, Veena A. Nair, Shruti Rajan, Poonam Beniwal-Patel, Sumona Saha, Vivek Prabhakaran

**Affiliations:** 1 Department of Radiology, School of Medicine and Public Health, University of Wisconsin-Madison, Madison, USA; 2 Department of Medicine, Division of Gastroenterology and Hepatology, Medical College of Wisconsin, Milwaukee, USA; 3 Department of Medicine, Division of Gastroenterology and Hepatology, School of Medicine and Public Health, University of Wisconsin-Madison, Madison, USA

**Keywords:** Cognitive neuroscience, Gastrointestinal diseases

## Abstract

Patients with inflammatory bowel disease have been shown to have abnormal brain morphometry or function, which are associated with psychological symptoms such as stress, depression or anxiety. The present work recruited 20 Crohn’s disease patients in remission (CDs) and 20 age-gender-handedness-education matched healthy controls (HCs) and compared their brain white matter microstructural properties using Diffusion Tensor Imaging (DTI). Additionally, we examined the correlations between the microstructural properties and cognition (verbal fluency language task, VF) and affect (anxiety) in both groups as well as disease duration in CDs. Results showed that CDs exhibited significant alterations in microstructural properties compared to HCs in various white matter tracts relevant to language function despite no significant difference in VF scores. Furthermore, CDs’ microstructural changes exhibited correlations with anxiety level and disease duration. These findings suggest that CD patients may experience changes in white matter microstructural properties which may be a biomarker of neuropsychiatric comorbidities of CD.

## Introduction

Crohn’s disease (CD), as one subtype of inflammatory bowel disease (IBD), is thought to be caused by disruption of the normal immune system and also perhaps by altered intestinal permeability^1^ and it can affect any gastrointestinal part from the mouth to the anus^2^. The impact of IBD may also extend to the brain and lead to anatomical and functional changes. Several studies have reported anatomical and functional brain changes in CD patients, possibly due to increased proinflammatory cytokines or chemokines and microglial cells which play an important role in communication between the brain, gut, and systemic immune system. These systemic changes have been posited to lead to a cascade of neuroplastic events that result in anatomical or functional brain changes that affect cognitive or affective abilities^3–5^. The observed brain changes might also account for the increased sensitivity of the CD patients to their related external environment which has been described among CDs and the inadequate ability to modulate cognitive and emotional states^3,6,7^. Moreover, the comorbidities assciated with IBD such as psychological stress, anxiety, depression, chronic pain may also influence anatomical and functional changes in the brain^1^.

A growing body of evidence suggests that abnormalities in the brain morphometry or function of CD patients may correlate with cognitive and affective changes. Several studies, in CD patients, have reported changes in brian morphometry compared to age-matched healthy controls (HCs). For exmaple, Bao *et al*. reported decreased cortical thickness in several regions and this decrease was correlated to pain score or disease duration^8^. Increased cortical thickness has also been reported as well as decreased sub-cortical volumes have been correlated to pain score or disease duration^3^; Zikou *et al*. showed decreased volume in the bilateral fusiform, inferior temporal gyrus (emotional processing), right precentral and middle frontal gyri (related to evoked stress responses) in CD patients^9^. Moreover, CDs with extraintestinal manifestations showed increased cortical surface area in the left middle frontal gyrus and hypergyrification in the left lingual gyrus (responsible for depression)^10,11^, while CDs without extraintestinal manifestations showed hypogyrification of the right insular gyrus and hypergyrification of the right anterior cingulate cortex (responsible for emotional processing such as grief, sadness, pain)^12^. These differences in results are possibly due to differences in patients age, handedness, disease characteristics and comorbidities, as well as sample size of these different studies. As to brain function, a task-based functional neuroimaging (fMRI) study with verbal fluency (VF) task showed CDs’ activity intensity in right hemisphere (key homologous) regions to be positively correlated with disease duration^13^; another study with a stress task consisting of Stroop color-word interference showed increased activity in the midcingulate cortex in CDs’ which possibly indicates an association between stress and symptomatic disease^14^; Another study showed increased sub-cortical activity (i.e. cingulate cortex, insula, amygdala, thalamus, hippocampus that correlate to trait anxiety and uncertainty intolerance) in CDs’ while responding to an uncertainty condition^15^. Moreover, a resting-state fMRI study in CDs’ showed abnormal connectivity within the default mode network (DMN) network (i.e. anterior cingulate cortex, superior medial frontal gyrus, middle cingulate cortex), which is known to be involved in processes such as internal monitoring, rumination, and self vs. other judgement^16^. Our previous resting state fMRI study of CDs’ showed significantly increased resting-state functional connectivity (RSFC) between the right middle frontal gyrus and right inferior parietal lobule as part of the executive control network (ECN – involved in working memory, reasoning, and in interactions with the external stimuli and environment) as well as increased RSFC between the right precuneus and right posterior cingulate cortex as part of the DMN^17^.

A recent study examined the brain white matter (WM) structure in IBD patients with diffusion tensor imaging techniques (DTI, as one non-invasive MRI technique for *in vivo* mapping of white matter structures that provides detailed information on underlying fiber tract architecture as reflected by diffusion patterns of water molecules^18,19^) and compared with their age-matched HCs^9^. Through whole-brain voxel level analysis, IBD patients (CD or ulcerative colitis) showed decreased WM axial diffusivity in the right corticospinal tract (involved in motor function) and right superior longitudinal fasciculus (involved in language function) when compared to HCs, indicating possible alterations in WM microstructural tissue properties in these patients. However, few studies have investigated the association between alterations in microstructural properties and deficits in cognitive and affective processing in IBD patients. Here, we used DTI to examine the alterations in microstructural properties in CDs in remission when compared to HCs and also examined the association between DTI metrics and cognitive and affective measures, as well as disease duration.

## Results

### Behavior

There were no significant differences between CD patients and HCs on age, handedness, education, and VF score (Table 1 shows participants’ basic demographic information).

**Table 1:** Differences of cognitive/neuropsychiatric measures and DTI metrics between participants of Crohn’s disease and healthy control.

Cognitive/neuropsychiatric measures/DTI index	CDs	HCs	*t*_(38)_	*p*
**Characteristics**				
Number	20	20		
Age (years)	35.85 (15.78)	33.60 (20.38)	0.39	0.69517
Education (years)	15.75 (2.63)	16.70 (1.92)	−1.30	0.20475
Gender (male/female)	12/8	12/8		1
Handedness (L/R/A)	2/15/3	1/19/0		0.13417
Mean VF score	43.30 (12.09)	42.30 (15.83)	0.22	0.81797
Mean BAS score	16.67 (1.84)			
Mean BIS score	22.26 (6.96)			
Duration of CD (years)	11.25 (8.74)			
IBD Medications	Antibiotics 0, 5- aminosalicyclate 7 immunomodulator 6, antitumor necrosis factorα 9, anti-integrin1, corticosteroids 0			
**DTI metrics**				
***FA***				
Left cingulum (cingulate gyrus)	1.11 (0.04)	1.16 (0.04)	−3.85	0.00044
Right cingulum (cingulate gyrus)	1.11 (0.03)	1.15 (0.04)	−3.57	0.00099
***MD***				
Left cingulum (cingulate gyrus)	0.98 (0.02)	0.96 (0.02)	4.07	0.00023
Left inferior fronto-occipital fasciculus	1.02 (0.01)	1.01 (0.01)	3.99	0.00030
Left superior longitudinal fasciculus	0.97 (0.01)	0.93 (0.02)	6.51	0.00011
Right superior longitudinal fasciculus	0.95 (0.01)	0.93 (0.02)	5.23	0.00050
***AD***				
Left cingulum (cingulate gyrus)	1.05 (0.03)	0.99 (0.03)	5.95	0.00067
Right cingulum (cingulate gyrus)	0.99 (0.04)	0.94 (0.03)	4.21	0.00015
Left superior longitudinal fasciculus	0.90 (0.02)	0.87 (0.02)	4.57	0.00004
Right superior longitudinal fasciculus	0.90 (0.02)	0.87 (0.02)	3.87	0.00042
***RD***				
Right corticospinal tract	0.87 (0.04)	0.83 (0.04)	3.69	0.00069
Right inferior longitudinal fasciculus	1.10 (0.02)	1.06 (0.03)	4.00	0.00021
Left superior longitudinal fasciculus	1.06 (0.05)	1.01 (0.04)	4.28	0.00012
Left superior longitudinal fasciculus (temporal part)	1.02 (0.05)	0.95 (0.06)	4.12	0.00020

Note: Standard deviations are shown in parentheses. All DTI metric values are less than *p* < 0.0025 (Bonferroni multiple comparison 0.05/20 tracts). CDs: Crohn’s disease; HCs: healthy control.

### DTI metrics

#### Group difference

**Figure 1:**
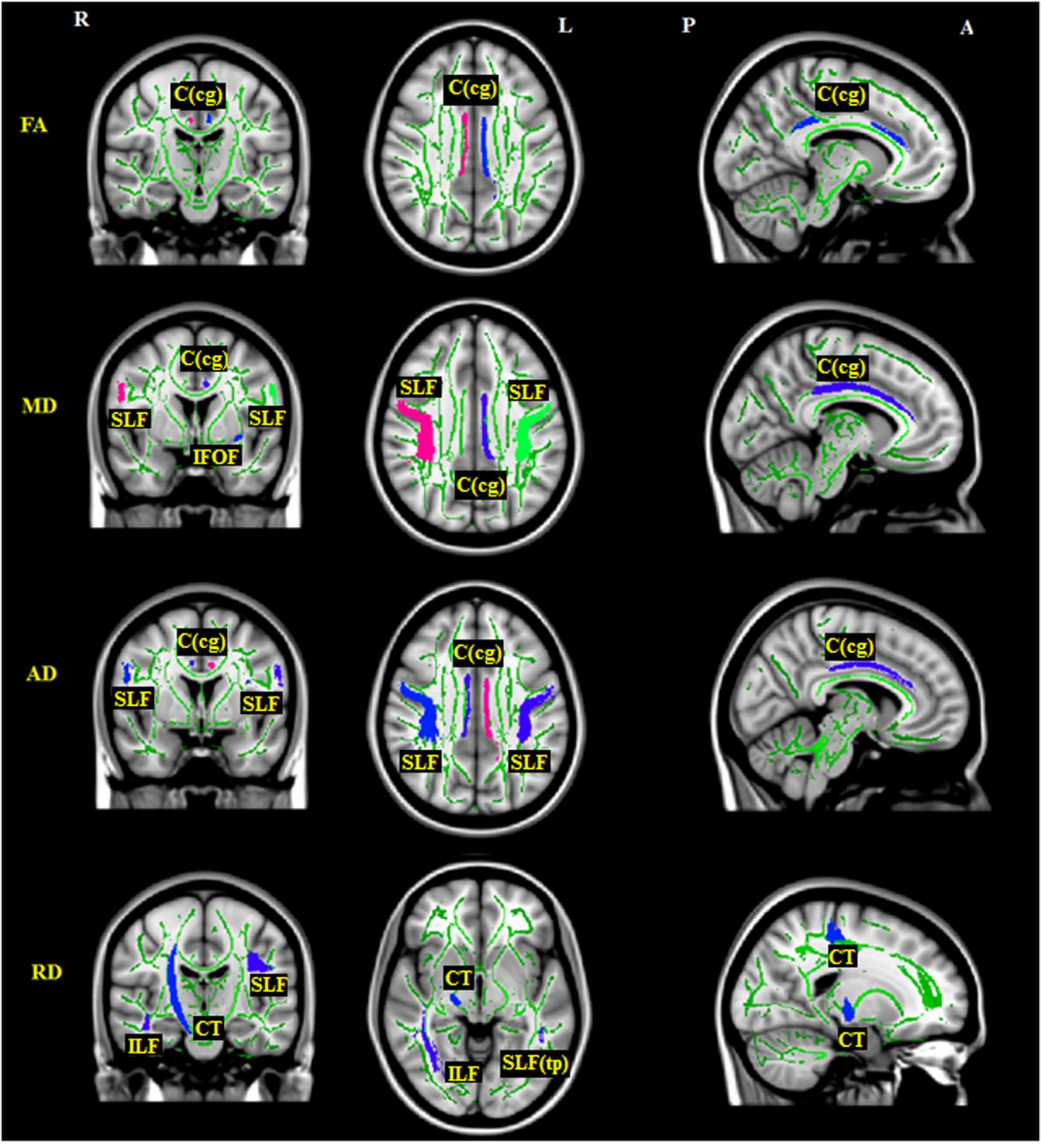
DTI metric differences between participants of Crohn’s disease and healthy control. R: right; L: left; A: anterior; P: posterior. C(cg): cingulum (cingulate gyrus); IFOF: inferior fronto-occipital fasciculus; CT: corticospinal tract; ILF: inferior longitudinal fasciculus; SLF: superior longitudinal fasciculus; SLF(tp): superior longitudinal fasciculus (temporal part).

Compared to HCs, CDs had: (1) significantly decreased fractional anisotropy (FA) values on regions of the bilateral cingulum (cingulate gyrus) [C(cg)]; (2) significantly increased mean diffusivity (MD) values on regions of the left C(cg), left inferior fronto-occipital fasciculus (IFOF) and bilateral superior longitudinal fasciculus (SLF); (3) significantly increased axial diffusivity (AD) values on regions of the bilateral C(cg) and bilateral SLF; (4) significantly increased radial diffusivity (RD) values on regions of the right corticospinal tract (CT), right inferior longitudinal fasciculus (ILF), left SLF, and left superior longitudinal fasciculus (temporal part) [SLF(tp)]. All details can be found in Table 1 and Fig. 1.

#### DTI metrics and cognitive/neuropsychiatric correlations

In HCs, VF score had significantly negative correlations to the left SLF and left SLF(tp) on the RD metric. However, CDs did not have significant correlations to any metrics. CDs’ anxiety scores had significantly negative correlations to the bilateral C(cg) on FA, to the right ILF and forceps major (FMa) on MD, to the FMa on AD, and to the right ILF on RD. Moreover, CDs’ disease duration was significantly positively correlated to FA values in the left anterior thalamic radiation (ATR), MD in the right IFOF, left SLF and left SLF(tp), RD in the forceps minor (FMi), as well as significantly negative correlations to FA in the right IFOF and FMi, MD in the right ATR, AD in the bilateral CT and FMi, RD in the bilateral ATR. All details can be found in Table 2.

**Table 2:** Correlations between DTI metrics and cognitive/neuropsychiatric measures.

Measures	Participants	Regions	FA	MD	AD	RD
VF	HCs	Left superior longitudinal fasciculus	—	—		−0.46*
		Left superior longitudinal fasciculus (temporal part)	—	—		−0.47*
Anxiety	CDs	Left cingulum (cingulate gyrus)	−0.50*	—	—	—
		Right cingulum (cingulate gyrus)	−0.45*	—	—	—
		Right inferior longitudinal fasciculus	—	−0.54*	—	−0.52*
		Forceps major	—	−0.52*	−0.45*	—
Disease duration	CDs	Left anterior thalamic radiation	0.57**	—	—	−0.47*
		Right anterior thalamic radiation	—	−0.54**	—	−0.63**
		Left corticospinal tract	—	—	−0.61**	—
		Right corticospinal tract	—	—	−0.58**	—
		Right inferior fronto-occipital fasciculus	−0.40**	0.44**	—	—
		Left superior longitudinal fasciculus	—	0.47*	—	—
		Left superior longitudinal fasciculus (temporal part)	—	0.50*	—	—
		Forceps minor	−0.64**	—	−0.53*	0.52*

Note: *p < 0.05, **p < 0.01.

## Discussion

### Group differences

Few studies have used DTI to study white matter tracts in CDs in remission. Our study suggests that brain white matter microstructural properties are significantly altered in CD patients. Our findings support the growing number of studies that have applied neuroimaging to examine the neural substrates impacted by the disease as well as its relationship to cognitive or affective processes^5^ and disease duration^3,8,20^.

FA, which quantifies the directionality of diffusion within a voxel between 0 (undirected, isotropic) and 1 (directed, anisotropic), is derived from the diffusion tensor and is the most commonly studied diffusion parameter of white matter microstructural properties related to the integrity of the fiber tract^21,22^. Increased (or decreased) FA in white matter tracts involved in cognitive processes is related to improved (or declined) cognitive function^7,23^, but the reduced FA in white matter tracts related to affective processes is related to increased severity of depression, anxiety or stress^24–26^. In the current study, the decreased FA on the bilateral C(cg) in CDs’ may indicate increased severity of depression or anxiety (the C(cg) is responsible for pain, depression, mood, and anxiety perceptions^16,27–29^). In contrast, MD characterizes the presence of obstacles to diffusion^30^. The increased MD indicates impaired fiber integrity and has been associated with reduced cognitive^7,31,32^ and affective functions^33,34^. In CDs, the increased MD in the left C(cg) may indicate increased stress, depression and anxiety in these subjects^35–37^. Increased MD in the left IFOF and bilateral SLF may indicate decreased language function (both the IFOF and SLF are involved in language function^22,38,39^). However, there was no VF performance difference between CDs and HCs, but these subjects showed a task-related compensatory fMRI activation as reported in our previous study which may have resulted in equivalent verbal fluency performance^2^.

AD measures the diffusion of water parallel to axons and primarily indicates axonal status^40,41^ by describing the principal eigenvector about the integrity of axons or the changes in extra-axonal and extracellular space^7,42,43^. The increased AD is generally associated with decreased cognitive ability^44–46^ as well as is associated with greater fatigue, pain, hyperalgesia^47^, depression and stress levels^48^. Therefore, the increased AD in the bilateral SLF in CDs’ could reflect the decrease in language function. The increased AD in the bilateral C(cg) may reflect increased pain, depression, mood or anxiety feeling, in CDs. In contrast, RD measures water diffusion perpendicular to fibers and mainly indicates myelin changes^40,41^. Like MD, the increased RD reflects decrease in cognitive function^49^ but with increase in depression or anxiety level^37,48^. The increased RD on the right CT may reflect decreased sensorimotor function (the CT is involved in sensorimotor function^50–52^). Increased RD on the right ILF, left SLF, and left SLF(tp) may indicate decline in language function.

### Correlations

In HCs, the VF score was negatively correlated to the left SLF and right SLF(tp) on RD metric. As mentioned above, the group difference results showed that the RD value in these two regions were significantly decreased in HCs than CDs, so their negative correlations to VF performance possibly reflect the increased language function in healthy controls. However, CDs showed no significant correlations between VF score and any DTI metrics, and there was no VF difference with HCs. Again, it is likely that while the disease specific mechanisms lead to significant DTI changes in various language tracts (increased MD) that would suggest decline in language function, some patients possibly adapt using compensatory mechanisms to match VF outcomes of HCs as shown by our task-related fMRI study^2^ as well as the DTI changes in various language tracts (increased AD). Future research should evaluate for medication effects and prior disease severity as well as try to elucidate the specific mechanisms by which CD may cause WM microstructural property changes.

The DTI metrics were all negatively correlated to the anxiety scores in CDs. First, the cingulum, as a part of the limbic system, includes the cingulate gyrus and the parahippocampus, and connects to the hippocampus^53,54^. Studies have found activity in the cingulum during anxiety-related testing^16,27,28^, such as in individuals with social anxiety disorder (SAD) during a fear task with anticipatory anxiety^27^, as well as increased resting-state functional connectivity in the anterior cingulate cortex with left superior medial frontal gyrus and middle cingulate cortex (middle cingulate activity showed a significant association with anxiety scores in CDs^55^). The cingulum is also activated in both normal and pathologic anxiety^27,56^. Second, MD and RD values in the ILF has been found to be negatively correlated with anxiety scores, which is due to the abnormal functional connectivity of the fusiform and inferior temporal gyrus with the amygdala and insula (regions responsible for emotional processing^57–62^) which is also seen in SAD patients^35^. Third, the FMa, which is located at the interface between crossing fibers from the splenium of the corpus callosum and ILF^63^, has been found to be associated with anxiety symptoms^64^.

There are also some significant correlations between disease duration score and DTI metrics, such as the left anterior thalamic radiation (ATR) (with FA and RD metrics) and right ATR (with MD and RD metrics), bilateral CT (with AD metric), right IFOF (with FA and MD metrics), left SLF and left SLF(tp) (with MD metric), and FMi (with FA, AD and RD metrics). Similar to our findings with disease duration, a study found that the ATR, CT, IFOF, ILF, SLF and its temporal portion, were associated with the duration of heroin use as well as with anxiety and depression scores^65^. The ATR, which connects the anterior and ventromedial nuclei of thalamus to the prefrontal cortex (including the anterior cingulate and dorsolateral frontal regions^66–69^), is mainly related to sensorimotor^70,71^ and also to executive function, working memory^72^ and the levels of anxiety and depression^73^. The FMi, a commissure pathway connecting the bilateral frontal regions^66^, is associated with sensory and auditory processing^74^ as well as emotional disorders^24^. Moreover, the FMi and ATR can affect attention-control skills^67^. Therefore, together with the functions of language and psychiatric symptoms mentioned above, CD duration may affect many neural functions.

### Limitations

This study was limited by a modest sample size. First, increasing the sample size would be particularly important to power the analyses for identification of neural correlates linked to behavioral performances in CD patients. Second, here we did not collect patients’ information about previous medication use which may have lasting neural effects that could have biased the results. Third, handedness can affect brain functional and structural organizations^75–77^, brain size^75^ and cognitive ability (i.e. language and attention^76^). Although there was no statistically significant difference on handedness in our participant groups, influence of handedness on brain white matter microstructural properties merits further investigation. Fourth, the white matter atlas used in this study focused broadly on the bilateral cingulum (cingulate gyrus), without specifically distinguishing between anterior versus posterior regions that are known to be involved in distinct cognitive processes. These specific regions can be investigated in a future study. Finally, the confounding variables of age of onset, disease chronicity, and their potential relationships with microstructural property changes, should be investigated. Future studies which longitudinally and prospectively assess white matter changes in patients with CD are needed to evaluate the potential impact of disease flares, surgery, and IBD medication use on these changes.

## Conclusion

This study highlights the utility of DTI in assessing for brain manifestations of CD. Our results suggest that CD patients in remission exhibit alterations compared to HCs in various white matter tracts (e.g. language, sensorimotor, attention, executive function, pain, depression, and anxiety feelings). They also indicate a correlation between white matter patterns and anxiety outcomes and disease duration. White matter tract connectivity may, therefore, serve as a biomarker of neuropsychiatric comorbidities of CD.

## Methods

### Participants

Twenty patients with CDs (12 males and 8 females, mean age = 35.85, SD = 15.78) and twenty HCs (12 males and 8 females, mean age = 33.60, SD = 20.38) participated in this study. All participants provided written informed consent. Participants were included in the study if they were at least 18 years or older. CDs were diagnosed based on endoscopy, histology or radiographic imaging. Patients were determined to be in remission if they had a Harvey Bradshaw Index^78,79^ score of less than three^78,80^. Results of an analysis by Vermiere *et al*.^80^ which examined the correlation between CDAI and HBI in assessing Crohn’s disease activity supported defining HBI remission as an HBI score <4 points. Exclusion criteria included pregnancy, co-morbid pain disorder unrelated to IBD, scheduled medications for treatment of pain, and contraindications to MRI. A 0–10 rating scale^81–84^ was used to record the intensity of pain experienced during the week leading up to the study visit. The Center for Epidemiologic Studies Depression (CES-D; 20 items) scale^85,86^ was used to evaluate symptoms associated with depression. HCs were free of any medical, neurological, or psychiatric disorders. Table 1 shows participants’ basic demographic information and disease-related information for the CDs.

All methods were carried out in accordance with relevant guidelines and regulations. All experimental protocols were approved by the Institutional Review Board (IRB) of the School of Medicine and Public Health, University of Wisconsin-Madison. Written informed consent was obtained from all participants.

### Behavioral data acquisition

Both CDs and HCs were administered the VF task outside the scanner. Anxiety data was obtained from CDs using well-validated tests described below.

#### Verbal fluency

We administered the phonemic verbal fluency (VF) task (the Controlled Oral Word Association Test, COWAT^87^) to test cognitive function. COWAT has been extensively used in both clinical and non-clinical populations on account of its face validity^88^, assessment of both verbal cognitive ability and executive control^89^, and high correlation with measures of attention, verbal memory, and word knowledge^90^. Participants were required to produce words beginning with the letters “F,” “A,” “S,” in three 1-minute trials, respectively. The total number of correct responses was used to quantify VF. Since CDs and HCs were age- and gender-matched with similar education levels, raw scores achieved on the task were used in subsequent analyses.

#### Anxiety

Behavioral inhibition system (BIS) and behavioral approach system (BAS)^91^ scales were administered by a questionniare to measure anxiety^92^. BIS and BAS scores were calculated for each CD participant and included 24 items rated on a 4-point Likert scale for 20 score-items and 4 fillers. Total scores for BIS (range = 7~28; 7 items) and BAS (range = 13~52; 13 items) were used to measure anxiety. While a mean BAS score was computed encompassing domains of reward responsiveness, fun-seeking, and drive, a total BIS score was computed as a measure of response to adverse events.

#### Disease duration

CD disease duration was obtained by patient self-report followed by verification with the electronic health record.

### MRI data acquisition

Diffusion-weighted images were acquired using a spin-echo based, single-shot echo-planar diffusion sequence lasting 10 minutes on a GE750 3 T MRI scanner. The specific MR imaging parameters were: repetition time (TR) = 9000 ms; TE = 66.2 ms; single average (NEX = 1); field of view = 256 × 256 mm^2^; matrix size = 256 × 256; in-plane resolution = 1 × 1mm^2^; 75 axial slices with no gap between slices and slice thickness = 2 mm; excitation flip angle α = 90°; 56 gradient encoded directions, *b* value = 1000 s/mm^2^. A high-resolution 3D T1-weighted BRAVO, IR-prepared FSPGR (Fast Spoiled Gradient Recalled Echo), MRI sequence with 156 axial slices was performed for each participant using the following parameters: TR = 8.132 ms; echo time (TE) = 3.18 ms, inversion time (TI) = 450 ms; field of view = 256 × 256 mm^2^; matrix size = 256 × 256; in-plane resolution =1 × 1mm^2^; slice thickness = 1.0 mm; excitation flip angle α = 12°.

### Data preprocessing

All diffusion data were processed using the “Pipeline for Analyzing braiN Diffusion images” (PANDA): a toolbox implemented in MATLAB

(http://www.nitrc.org/projects/panda/)^93^. This software employs several neuroimaging processing modules including the FMRIB Software Library (FSL), the Pipeline System for Octave and Matlab (PSOM), the Diffusion Toolkit, the MRIcron to automatically perform a series of steps (i.e., skull removal, correction of eddy current distortion, build diffusion tensor models)^93^.

Tract-Based Spatial Statistics (TBSS) were employed to evaluate voxel-based group differences for the values of fractional anisotropy (FA), mean diffusivity (MD), axial diffusivity (AD), and radial diffusivity (RD)^93–95^. TBSS involves constructing a skeleton from the mean metric values of all aligned images following normalization, and subsequently projecting the obtained FA, MD, AD, and RD values from each participant onto this skeleton. Diffusion metrics for each participant were extracted for 20 tracts identified from the Johns Hopkins University (JHU)-ICBM-DTI-81 white matter atlas^93^. Each global mean metric (FA, MD, AD, RD) for each participant was obtained by averaging across the 20 tracts, and diffusion metric for each tract was divided by this global mean to account for any variability between participants, and the standardized metric was used in further statistical analysis.

### Statistical analysis

Two sample t-tests were performed to investigate group differences on each DTI metric, and the statistical threshold was considered the Bonferroni *p* < 0.0025 (0.05/20 tracts) as multiple comparison. Significant DTI metrics identified in the t-test were used to perform exploratory correlation analyses with VF task scores based on Pearson’s correlation using IBM SPSS version 23, and considered significant at *p* < 0.05. Correlations between DTI metrics and scores of anxiety and disease duration were computed only in CDs, because these data were not collected in HCs.
